# Rapid Video Responses (RVR) vs. Face-to-Face Responses by Police Officers to Domestic Abuse Victims: a Randomised Controlled Trial

**DOI:** 10.1007/s41887-022-00075-w

**Published:** 2022-05-31

**Authors:** Stacey Rothwell, Kent McFadzien, Heather Strang, Graham Hooper, Alan Pughsley

**Affiliations:** 1 Kent Police, Kent, UK; 2 Cambridge Centre for Evidence-Based Policing, Cambridge, UK; 3 Institute of Criminology, University of Cambridge, Cambridge, UK

**Keywords:** Domestic abuse, Differential police response, Caller satisfaction with police, Rapid video response, VAWG (violence against women & girls)

## Abstract

**Research Question:**

Can police increase victim satisfaction and improve efficiency by providing consenting domestic abuse victims, *if their offenders are not present*, with an immediate video link to a uniformed police officer, rather than waiting for face-to-face police attendance?

**Data:**

Eligible and consenting cases for this block-randomised trial (*N* =517) included 357 calls from female victims of intimate partner violence (FIPV), 57 calls from female victims of non-IPV abuse, 83 calls from male victims of IPV and 21 calls from male victims of non-IPV abuse. Cases were screened for eligibility before police call takers asked all callers for consent to a video meeting with a police officer (if one was available for random assignment).

**Methods:**

Consenting callers (560 of 749 of eligible callers = 75%) agreed to be randomly assigned to either ‘business as usual’ (BAU) for such calls or a rapid video response (RVR), by immediate transfer of their call to a uniformed police officer visible on a video link (to whom the victim was also visible); only 69% (517 of 749) were randomly assigned due to limited RVR availability. Follow-up interviews with all 511 assigned callers consenting to interviews (99% of those assigned) were completed with 80.5 % (*N* = 416) of those callers across both treatment groups, with 82.2% in RVR and 78.7% in BAU, and near identical rates for female IPV victims as for the combined samples. Official records on arrest and other short-term case outcomes were also collected. Data were analysed in four blocks of separate random assignment sequences for each of the categories of callers. Across all four blocks combined, 94% of the RVR-assigned victims received a completed RVR resolution; 67% of the BAU victims received a face-to-face contact with a police officer, with other BAU responses by voice phone but not video.

**Findings:**

Rapid video response (RVR) to these calls was an average of 656 times faster in responding to the victims (3 min) than the average BAU time for trying to deploy a police car (1969 min). RVR clearly produced higher victim satisfaction among female IPV victims (89% in the RVR group) compared to control victims (78% in the BAU group) (*p*=0.01). Arrest rates for suspects were 50% higher in the RVR group (24%) relative to the BAU group (16%), with three times more arrests during follow-up investigations on RVR cases. Trust and confidence in the police improved more for abuse victims receiving RVR than those receiving BAU.

**Conclusions:**

In this test, RVR helped domestic abuse victims far more rapidly than BAU. The innovation improved domestic abuse victims’ satisfaction, and their trust and confidence in the police, who made more arrests after RVR than BAU. Based on these conclusions, Kent Police have launched RVR county-wide as a new standard offering for domestic abuse victims to select.

## Introduction 

Since the advent of radio-equipped police cars in California in the 1920s (Oliver, 2017: 416), there has been a relentless increase in public demand for police to drive to their homes as soon as possible, often to the exclusion of other ways for police to help people. By the 1980s, it became clear that alternatives to dispatching police cars were needed for dealing with police matters. Such alternatives were seen to be needed for both the convenience of callers and the efficiency of police resources.

Some of these alternatives were field-tested in three US cities, with encouraging results (McEwen et al., 1986). Telephone reporting of crimes, for example, which was included in that successful test, became a widely reported practice in many police agencies in many countries. Yet for domestic abuse, the demand for rapid, in-person response only intensified after the Minneapolis Domestic Violence Experiment (Sherman & Berk, 1984). This demand was linked to the aim of mandatory arrest of the offender at the scene, which could only be accomplished with rapid police response by at least two officers.

That ‘positive action’ policy, as arrest for domestic abuse was called in the UK, rested on the assumption that suspects would still be on the scene when police arrived. Yet, in the Milwaukee experiment, most offenders were still at the scene when police arrived, which they did within a mean of 30 min after the victims’ called (Sherman, 1992: 327). That response time is far shorter than has been possible in twenty-first century UK. A recent study in one area of London (Richards & Harinam, 2020), for example, found that in 71% of a sample of 1000 domestic abuse cases, offenders had left the scene by the time police arrived.

In the case of Kent Police, many domestic victims were known to be calling police after the abuser had left the scene of the abuse (which was usually the victim’s home). Yet, in the absence of an assurance that the suspect would not return, prudence required that two officers should respond together to such calls. At the same time, a steep rise in the numbers of these calls meant that the time to arrival kept increasing.

Based on a previous, successful 2020 experiment (Rothwell, 2020) with voice-only telephone communication (launched before the COVID Pandemic) with *non*-domestic crime victims, Kent Police chose in 2021 to test the use of visual communications media for immediate contact between abuse victims and experienced police officers. The randomised controlled trial (RCT) was to be limited to cases in which *abuse suspects or victims have already left the scene*, and for which victims gave their consent to receive their police response by digital media rather than in person. From 23rd February to 28th May, 2021, police call takers in one control room screened and randomly assigned 517 such cases to either the current ‘Business As Usual’ (BAU) practices for such cases or rapid video response (RVR) by a uniformed police officer.

In order to achieve more precision in assessing the impact of the RVR innovation on different kinds of domestic abuse victims, Kent Police agreed to have the Cambridge Centre for Evidence-Based Policing create four separate tests within the overall experiment. In each of these tests, the random assignment sequence was separated to make them, in effect, four separate experiments, one for both males and female callers who reported that they had been abused by either an intimate partner or a family member.

In this article, we report results from both the combined findings of all four ‘block—randomised’ RCTs (Ariel & Farrington, 2010) and from the female intimate partner violence (FIPV) victims separately. The latter group comprised some 7 out of 10 cases in the experiment, and the greatest statistical power of the four blocks for measuring the effects of RVR vs. BAU policing with offender-absent domestic abuse.

## The Setting: Kent and UK Police

Kent Police is a large county force in the southeast of the UK. It protects and serves an estimated population of 1.87 million across metropolitan centres, towns, villages, and rural hamlets with approximately 7000 police officers and civilian staff. Kent Police is broadly representative of the national landscape for domestic abuse service provision. HMICFRS inspections have found delays in attendance to domestic abuse calls, in a quarter of police forces and attributed this, in part, to a lack of officers and rising numbers of such calls.

At the same time the increasing delays were documented, the National Police Chief’s Council (NPCC) drafted a new contact management strategy. The plan directs police forces to provide an intelligent response to victims at the earliest point of police contact utilising technology, offering alternatives to physical attendance (NPCC, 2019). The possibility for such alternatives increased substantially in early 2021 when Kent Police developed an enhanced video communication strategy in response to the COVID pandemic. The availability of this new technology enabled Kent to do exactly what the NPCC was calling for. Moreover, Kent became able to target this innovation for supporting violence against women and girls (VAWG), about which the Home Office (2021) was also calling for new strategies. This opportunity provided the ideal conditions for evidence-based policing, based on an impact evaluation from randomised controlled trials with four different kinds of domestic abuse victims.

## Research Questions

The four trials of RVR services for domestic abuse were designed to answer the same set of questions. While it was anticipated that some of the samples would be too small for adequate statistical power, the design allowed the questions to be answered for all groups combined—as well as for the largest subgroup, which we expected to be around 70% of the entire sample.

Given the context of developing alternative responses to domestic abuse, our overall research question was this:

*Can police increase caller satisfaction and improve police efficiency via uptake of an optional video response (RVR) provided immediately to suitable mid-level domestic abuse callers.*

This question encompasses several sub-questions related to caller satisfaction from RVR, when compared to business as usual (BAU), as follows:

1. What is the impact of RVR on police ‘response’ times to establishing contact with the domestic abuse victims?
2. What is the impact of RVR on police ‘resolution’ times in working with the domestic abuse victims?
3. What are the eventual outcomes of incidents reported to police via RVR, including arrests and criminal offence reports?

Overall, Kent Police wanted to know if offering RVR could improve their service to victims in a way that was just as, or more, efficient and effective as current response options.

## Two Response Protocols: BAU vs. RVR

Rapid video response (RVR) is a new virtual policing *response* option. It serves as a first response to calls for service from members of the public who contact police by calling the emergency telephone numbers (999 and 101). Eligible victims can speak with an officer over video and get a service commensurate with what would take place were police to visit the victim’s physical location. This includes the reporting of any crimes, receiving safeguarding advice and assistance and allowing police to complete relevant risk assessments and investigative steps required to advance potential criminal proceedings. Any investigative or safeguarding steps that police are unable to complete over a video call can be tasked to be completed by other officers either immediately or at a future point in time (a decision based on risk). As a response option, officers are ‘dispatched’ virtually to speak with victims to gather a full understanding of what has happened. It is not a resolution service that is provided to victims where police already know what has happened and are simply allocating resource to resolve it.

As an example of how RVR works, the authors have produced a fictional short video clip, showing both a victim and a police officer, to portray a fairly typical dialogue. The video is available at this link: https://www.cambridge-ebp.co.uk/videos.

This section outlines where RVR sits within an operational context. It begins by describing the standard, BAU response from the victim’s perspective, as well as by the broader police perspective. It then describes the RVR process by explanation and comparison, with key differences highlighted. RVR was applied only to mid-level domestic abuse calls for service if the offender was absent. This means that it has been tested on a subset of potentially suitable calls and callers for a wide range of policing issues. RVR in this context could even be more accurately described as ‘domestic abuse rapid video response’, or ‘DA-RVR’, but in this article, we simply call it RVR for brevity.

### Business as Usual Process

#### Call Taker Phase

When reporting domestic abuse, victims who called 999 or 101 are connected to a police staff employee in the Kent police force control room (FCR). The staffers discuss the incident with the caller, during which staffers assess whether or when a victim needs to see an officer. This ‘THRIVE’ assessment process considers the six dimensions of threat, harm, risk, investigative requirements, vulnerability and engagement. Through this framework, police call takers are instructed to grade each call across five substantive categories each with their own individual processes for responding. These categories are:

1. *Immediate response cases*: where the call record gets passed rapidly to a dispatcher who sends a patrol car.
2. *Priority cases*: where the call record is classified as having circumstances that while no immediate response is required, there is a need for police to attend in a timely manner.
3. *Appointment*: the incident can be appropriately dealt with via a booked appointment in the coming few days, and can be at the victim’s home address (or another safe address), or at a police station.
4. *Taskings* and other types of response: this resolution is generally for calls about previously reported incidents. It may involve updating officers in charge of the case about new information or the requirement for completing other investigative functions.
5. *Resolving without deployment* (RWD). While it is rare for domestic abuse incidents to be graded RWD in Kent, this final category is for incidents that can be resolved without any officer travelling to meet the victim face-to-face.

RVR is designed for mid-level domestic abuse, specifically categories #2 and #3 above (priority and appointment calls). The decision for experimental design was that the highest- and lowest-risk grades are not appropriate for a video service. In the highest, victims require immediate physical police attendance to protect the victim; at the lowest (taskings and RWD, #4 and #5), no physical attendance is needed.

#### Police Officer Response Phase

While the route to attendance for immediate-, priority- and appointment-graded calls differs slightly, Kent Police provide a uniform initial response to victims on attending to their call. This is generally done in person and involves the officer recording and investigating any criminal offences that have been committed and collecting appropriate evidence; they also carry out the domestic abuse risk assessment and provide appropriate safeguarding and referrals to partner agencies. At the conclusion of this phase of the case, the primary investigation is completed, and the officer leaves the scene (or victim leaves the station). The case then gets passed to Kent Police’s specialist domestic abuse investigation teams who continue investigating, completing the secondary investigation and undertaking further safeguarding as required.

#### Victim Perspective

Many of the actions of police go on behind the scenes from the perspective of the victim, who chose to call police and have a discussion with a call taker. Victims are then advised of what service they will be receiving. For priority calls, this involves being told to *wait where they are until the police arrive and call back if circumstances change*. They are generally not told to expect a specific arrival time, but simply that a police car will arrive as soon as possible. For appointments, they will have a choice of the time and location; if no suitable appointments can be arranged in a timely manner, considering the level of risk, then the call could be changed to a priority grading and the victim told to wait for attendance. In both cases, the call ends with the victim expecting a service after a period of delay. This delay is anticipated to be either short but of an unknown duration (priority) or (by appointment) longer, often a couple of days, but at a fixed time.

Once the victims engage with the police officer(s), they are asked to explain what has happened and are provided with support and safeguarding advice. They are also provided with information about what will happen next, such as arrest of the suspect or the expectation of an ongoing investigation. If appropriate, an evidential victim statement will be taken and other evidence captured.

### Rapid Video Response: the RVR Experimental Process

**Fig. 1:**
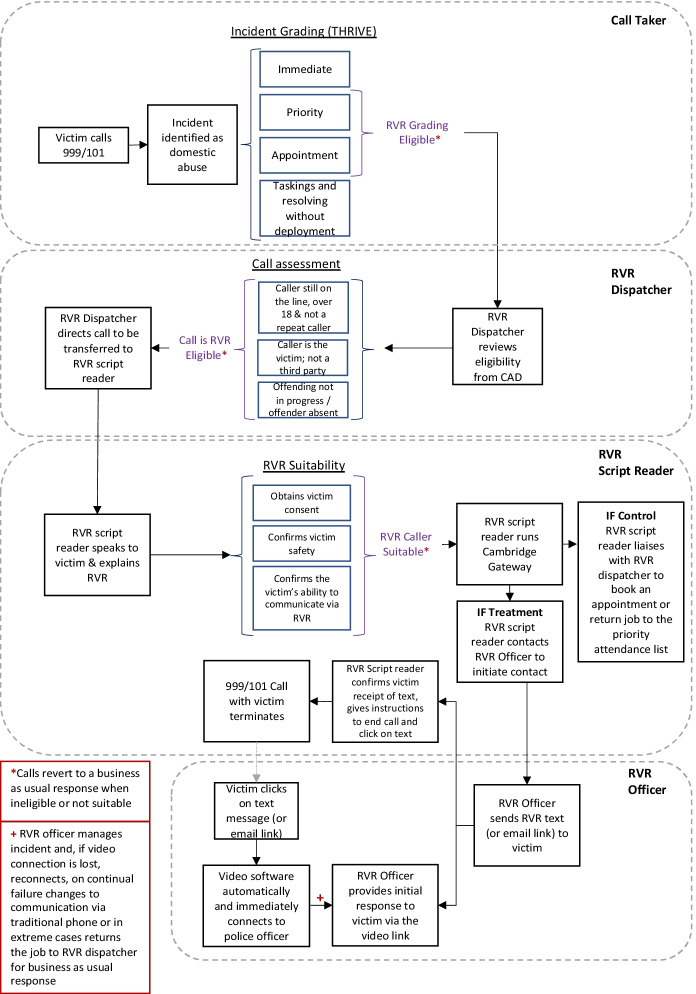
Rapid video response process

The full RVR experimental process is mapped in Fig. 1, for both the victim and the police. It alters the standard BAU process in several ways. From the perspective of the police, it introduces the requirement for a new dispatcher. Under the standard model, calls are transferred through to dispatchers who have oversight of different geographies. The RVR dispatch ‘region’, in contrast, is ‘virtual’ and sits alongside these geographic dispatchers. It is a specialised role requiring the review of ongoing mid-level priority and appointment domestic abuse calls that call takers are on. The dispatcher reviews these to make sure that the basic eligibility criteria for RVR are met:

1. The call is identified as domestic abuse and is graded ‘priority’ or ‘appointment’.
2. The victim is still speaking with the call taker.
3. The caller is over 18 and is the victim (not a third-party caller).
4. The caller has not been part of the randomised trial previously (for the trial only; a repeat caller policy was planned to be in place for RVR in a non-trial setting).
5. The suspected offender is absent at that time and the offending is not ongoing.

If these criteria were met, the RVR dispatcher communicated with the call taker by text messaging on the computer-aided dispatch (CAD) system that the case might be eligible. On completion of all the requirements, the call taker would then advise the victim that they may be able to have an immediate video response and that they will be spoken to by a colleague. This role was fulfilled by the script reader during the trial who managed the case flow and eligibility for the trial (see script at Appendix 1).

The victim was then transferred to a script reader (the trial-specific role) who read a script to the victim and discussed the RVR option, explaining what it is and how it works. They also assessed whether the victim is safe and can communicate. Victim safety is primarily victim led with the victim asked to confirm if they are in a ‘safe and private place’. During the trial, an added layer of oversight was employed with a specialist domestic abuse sergeant overseeing all CAD for safety. When those conditions were satisfied, the script reader then offered victims the opportunity to opt in to the RVR service.

The ability to communicate had to be established on several dimensions. First, the victim had to be freely able to communicate over video with appropriate technology and signal, and the location had to be quiet, private and not under surveillance by the accused perpetrator. The victim must also not have been intoxicated, having a mental health crisis or other conditions that inhibited the free flow of information between an officer and the victim. Finally, victims had to consent to their video meeting with the police officer (if available) being recorded for potential use as evidence in a prosecution.

## RVR Trial Design

### 4-Block Structure

The RVR trial had a block randomised design, using blocking based on victims’ gender and their relationship to the perpetrator (see Appendix 2 for overview of caseflow and decision pathways). This led to four blocks of consenting victims, each of whom was randomised into one and only one of the blocks:

- Female victim whose perpetrator is or was an intimate partner (FIPV)
- Male victim whose perpetrator is or was an intimate partner (male intimate)
- Female victim whose perpetrator is a non-intimate family member (female non-intimate)
- Male victim whose perpetrator is a non-intimate family member (male non-intimate)

Intimate partners included both current and ex-partners. Non-intimate partner violence categories include other intra-family harm, such as violence from siblings, parents or children.

**Fig. 2:**
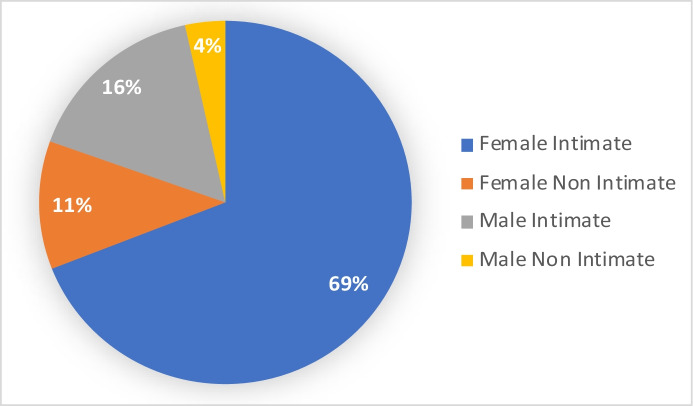
RVR trial participant categories (*n* = 517)

This design was chosen to increase the precision of the analysis by grouping the experimental subunits so there is less variability between members of the same subset than members of the different subsets. Most importantly, it allows specific analysis of female intimate partner victims who were by far the largest subgroup and are of professional and academic interest (Fig. 2).

### Time Frame

The RCT ran in Kent Police between the 23rd of February 2021 and the 28th of May, 2021. It incorporated 73 shifts: 68 weekday late shifts that ran from 2 to 10pm (with calls received until 9pm) and five Saturday daytime shifts starting at 10am and finishing at 6pm (calls received until 5 pm) (Table 1).

**Table 1:** Distribution of RVR case across the week

Day	Shifts	Cases	Average
Monday (1400–2200)	13	100	7.69
Tuesday (1400–2200)	14	102	7.29
Wednesday (1400–2200)	14	106	7.57
Thursday (1400–2200)	14	95	6.79
Friday (1400–2200)	13	83	6.38
Saturday (1000–1800)	5	31	6.20
Total	73	517	7.08

These eligible calls received their service intention at the point of allocation (with a grading as either ‘priority’ or ‘appointment’ calls). Both arms of the overall trial had an equal proportion of priority and appointment calls at 43% and 57%, respectively.

#### Treatments As Delivered

**Fig. 3:**
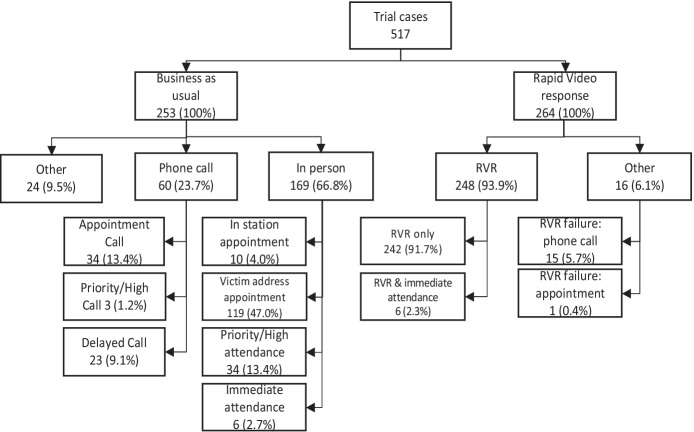
Service actually provided to callers after random assignment in the RVR trial

The service Kent Police ultimately provided to domestic abuse victims in the trial is shown in Fig. 3 below. A full CONSORT diagram appears at Appendix 3.

##### RVR-Assigned Cases

The RVR service is comparatively consistent, with the vast majority of those randomly assigned to it (93.9%) actually getting a rapid video response service. One case did not receive the RVR service getting an appointment instead; this was due to the victim knowing the only available RVR officer (This fact was not detected until after randomisation). A small number of cases received a mixture of RVR and telephone call due to technological challenges, usually an unsatisfactory internet connection (5.7%). There were a further 22 callers who received an RVR service of 15 min or less, with no mention by officers that they supplemented it with a phone call; these are included as standard RVR calls. Another small proportion of RVR-assigned victims received an elevated in-person service as circumstances changed during the RVR call requiring a patrol car to be dispatched (2.3%). These are deemed an immediate attendance even though they, like all calls, were initially assessed as having ‘priority’ or ‘appointment’ level risk. When the circumstances changed, it was most commonly when the offender returned while the RVR officer was communicating with the victim.

##### BAU-Assigned Cases

The business-as-usual cases showed much more variability between initial and eventual outcome than the RVR-assigned cases. This was probably due to the passage of time that had passed and its impact on risk. As time went by between the initial assessment and servicing of the call, dispatchers and officers made discretionary decisions altering how the call was ultimately serviced. This meant that 66.8% got an in-person response and 33.2% were spoken to over the telephone (Fig. 3). There were six immediate attendance calls and much like those of RVR discussed above these were initially graded priority or appointment and were elevated due to a change in circumstances, such as the return of the offender.

### Survey Methodology

Victims involved in the RVR trial were asked if they consented to a survey immediately after the point of random assignment. If they agreed, they were contacted by interviewers from the Cambridge Centre for Evidence-Based Policing. Attempts to contact them began 10 days after the call for service was randomly assigned to RVR or BAU. If this initial attempt failed, further attempts were made at varying times and days over several of weeks. A maximum of 13 attempts was made for each victim, with attempts stopping at either the 13th or if the victim answered and then declined to participate.

There was no requirement for victims to consent to the survey to be eligible for the trial. In total, 517 cases entered the trial for 514 individuals. Three individuals were erroneously entered into the trial twice and surveys were attempted for their first entry only. All three were FIPV cases: one refused to take part in any survey having received BAU followed by RVR, the other two were assigned to RVR on both of their entries into the trial and they were successfully surveyed about their first (but not second) RVR experience.

**Fig. 4:**
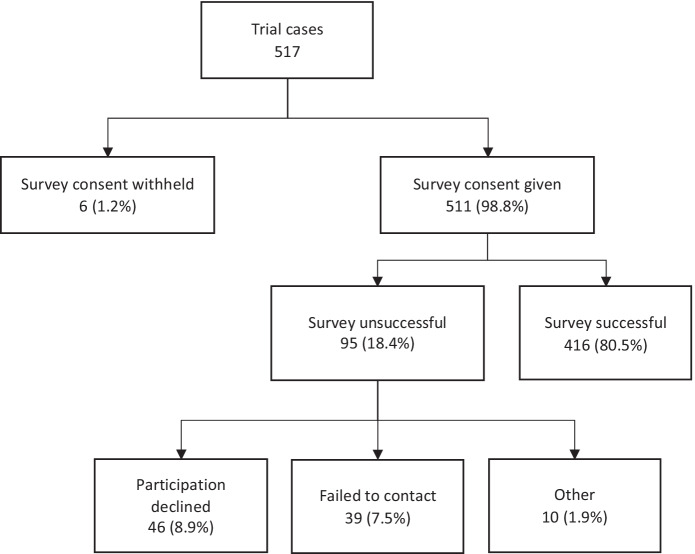
Attrition in victim survey participation

Participation by victims in the survey was high (Fig. 4). Victims were asked twice if they consented to taking part in a follow-up victim survey. The first request was post-random assignment, prior to allocation to an RVR officer or returning them to the business-as-usual process.^1^ At this point, most of the participants consented; there were only six callers (1.16%) who refused. Their details were not passed to the interviewers. Survey consent was reaffirmed by the interviewers when calling the victim back after 10 days. A further 95 victims dropped out at this stage with 46 (8.9%) declining and contact was not made with 39 (7.5%). A small number, 10 (1.9%), had survey attempts stopped due to the intervention or presence of the offender during an attempt to survey (3), were the second incidents of repeat callers (3) or stated they were not the caller (4). This last group who stated that they were not the caller still spoke with the call takers and received the appropriate police service the same as everyone else; they simply did not take the initial step of calling police. This decision to call police was made by a present third party. They have been excluded as it is unclear if they would have called police on their own volition. The overall participation rate in the victim survey was 80.5%.

## Findings

All analyses were undertaken based on the ‘intention to treat’ method. A total of 264 victims were assigned RVR and 253 assigned business as usual. These two groups were compared based on random assignment, irrespective of what victims in each case ultimately received. An overall analysis was supplemented with an equivalent analysis of the female intimate partner (FIPV) block. (Other blocks have not been analysed to date.)

### Victim Characteristics

#### Age

Most victims who called were aged between 25 and 44. No victim under the age of 18 was accepted onto the trial (this was an eligibility criteria). There were 18 participants aged 65 or older.

#### Ethnicity

A total of 407 victims reported their ethnicity to the interviewers with 85% of these (344) considering themselves white British.

#### Disabilities

A large minority of survey participants said that they had a disability. Of the 408 who responded, 131 (32%) answered ‘Yes’ to this question. The largest self-declared disabilities were anxiety (33), depression (21), ‘mental health’ (19) and PTSD (9). One of them told the interviewer that ‘[The video] conversation saved me having to sit there and wait for them or go to [the] station – I am disabled so I think it’s good for people who are in my kind of situation.’

### Victim Views

The victims were asked a broad range of questions about the service they received, their views of Kent Police and future service preferences. A selection of these items is reported here as comparisons between the cases assigned to BAU vs. RVR. Specifically, the comparisons include changes to victim anxiety, satisfaction, trust and confidence and their view of the impact of any delay in receiving their response.

#### Anxiety Change

**Fig. 5:**
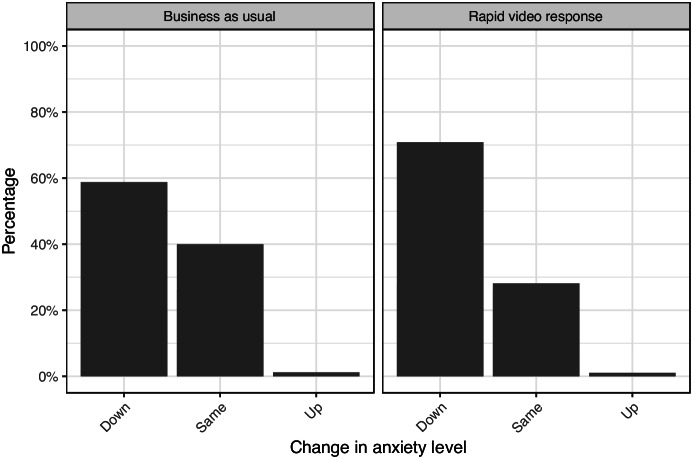
Change in self-reported anxiety for those initially reporting being very or quite anxious

With different treatments offered, the change in anxiety can be compared between those who received RVR and those who received business as usual. Of particular interest is the change in anxiety by those who initially reported feeling anxious. Figure 5 shows the anxiety levels at time of survey (at least 10 days after their call for service) of those who reported their initial anxiety to be ‘very anxious’ or ‘quite anxious’. Most of both groups report a decrease with over 70% of RVR respondents reporting a decrease in anxiety compared to almost 60% of BAU respondents. This is positive for both service offerings; however, the immediacy of RVR does offer the possibility of reducing victim anxiety faster. For the female IPV respondents who were assigned RVR, the anxiety decrease was even higher, with 75.9% reporting a decrease in anxiety compared to only 60% of FIPV assigned to BAU reporting a decrease.

#### Victim Satisfaction

**Fig. 6:**
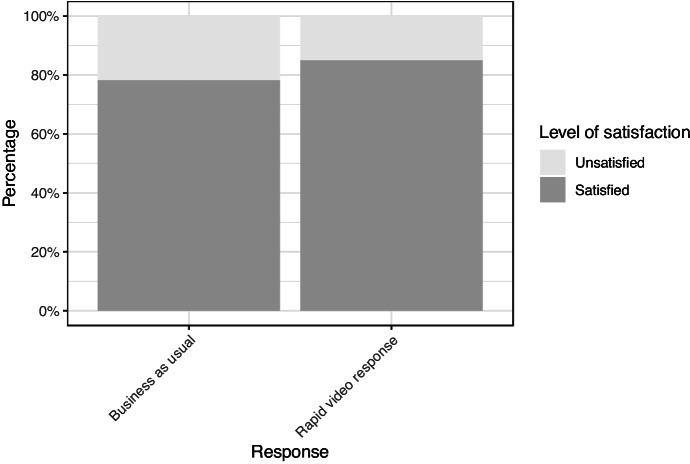
Overall satisfaction for all four blocks of participants combined

Victims were asked how satisfied they were with the initial service they received, whether that was business as usual or the new rapid video response (Fig. 6). The overall satisfaction for current practice is high, with 78.4% of business-as-usual respondents satisfied with the service they received. The satisfaction level of those who received the rapid video response was slightly higher overall at 84.9% (*X*^2^ (1, *n* = 402) = 2.84, *p* = 0.09).

#### Female IPV Victim Satisfaction

**Fig. 7:**
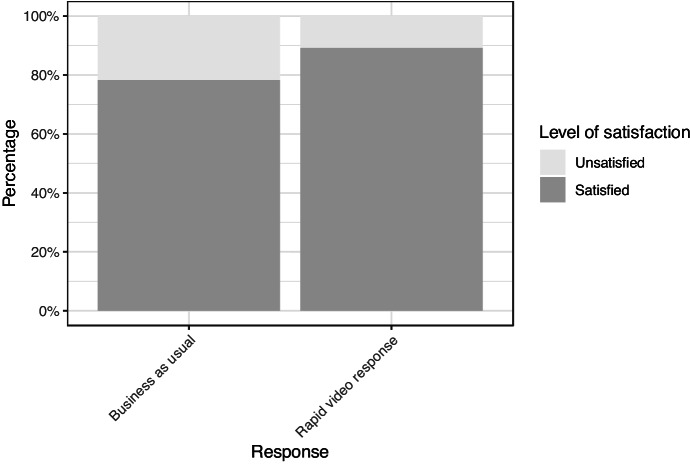
Satisfaction level with initial response for female IPV participants

The female intimate partner violence participants were by far the largest cohort and their number allows for analysis of this group separately. This allows RVR satisfaction to be assessed in the context of violence against women and girls. Figure 7 shows satisfaction of this block-randomised group at 78% for the BAU group vs. 89% for RVR. The statistically significant difference in favour of RVR in this group supports the conclusion that RVR works better than business as usual for this largest category of domestic abuse victims (*X*^2^ (1, *n* = 277) = 6.12, *p* = 0.01).

**Fig. 8:**
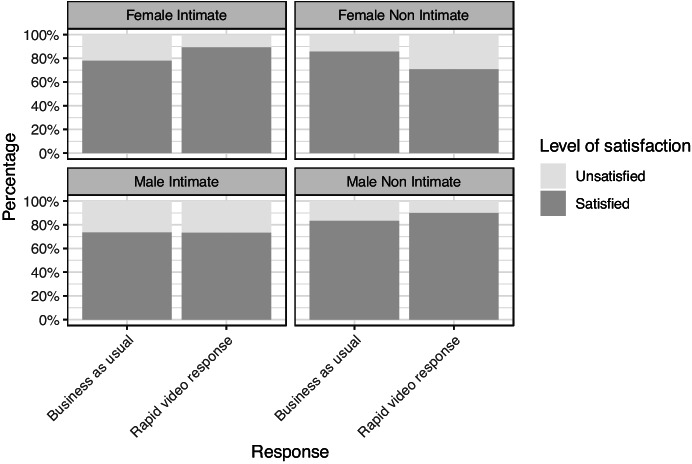
Satisfaction levels across the four block-randomised groups disaggregated

It is worth pointing out that in the female non-intimate group, satisfaction is lower than their BAU counterparts, at 71 versus 86%. That finding, however, is based on a much smaller sample of 45 female non intimate survey participants. Since only ten victims were unsatisfied (3 in BAU and 7 RVR), it is hard to draw any strong conclusions. Statistically, there is no evidence of any difference between the groups.^2^ Further research, with many more cases of non-IPV abuse of females, would be required to draw firm conclusions either in favour of BAU or RVR (Fig. 8).

#### Time Delay

**Fig. 9:**
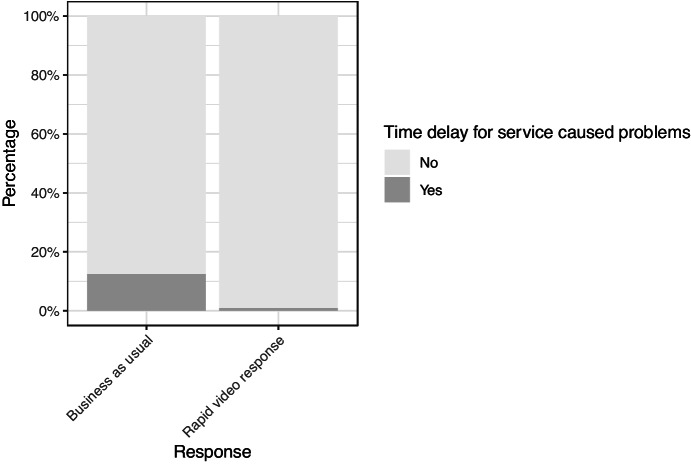
Percentage of participants who said the length of time they waited for service caused them problems

Figure 9 shows that victims were much more likely to report that the time it took to speak with an officer caused them problems in the BAU group than in the RVR group. Of the 405 responses, the vast majority said ‘No’, with only 26 participants who said ‘Yes’. This was unevenly distributed with 24 in the BAU group (12.5%) versus 2 in the RVR group (0.9%). Some of the comments for why this was the case included: ‘I was stressing and worrying and having to keep calling them back’, a second participant told an interviewer that a ‘second incident occurred’.

#### Impact on Confidence in Kent Police

**Fig. 10:**
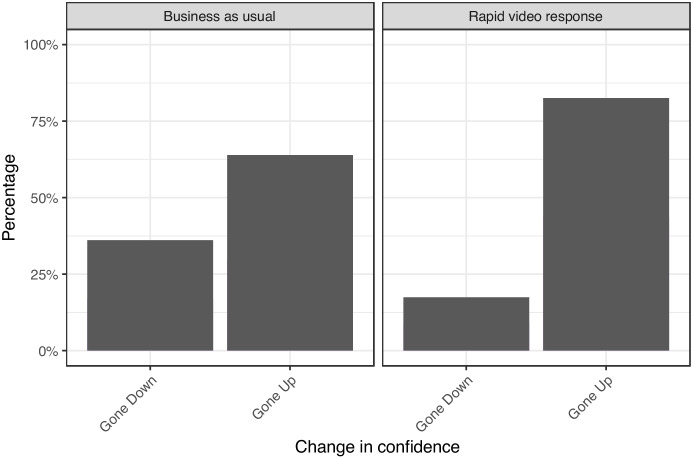
Victim change in confidence after police response

Interviewers asked victims how their trust and confidence changed as a result of the police response to their incident, if at all. Starting with changes in confidence, shown in Fig. 10, the graphic excludes the 48% of participants who said that their confidence did not change because of the interaction. The figure shows that 75% of RVR participants who reported a change in confidence reported that their confidence increased versus a 66% rise for BAU participants surveyed.

#### Impact on Trust in Kent Police

**Fig. 11:**
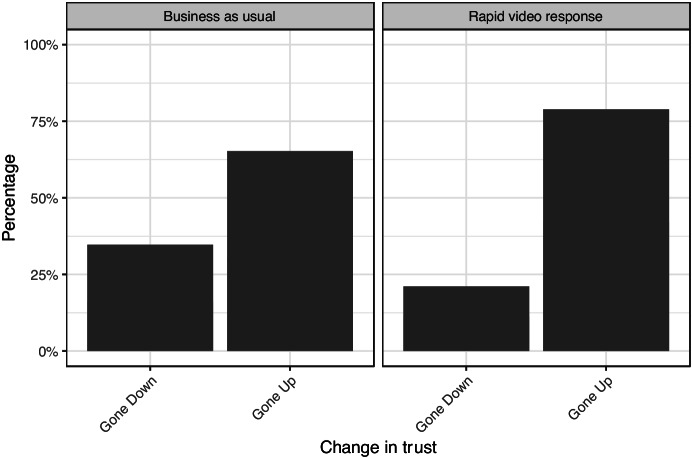
Change in trust in Kent Police by treatment

The changes in victims’ trust in Kent Police look broadly like the changes in confidence. Over 75% of RVR participants who stated that their trust changed reported an increase versus two-thirds of business-as-usual participants (Fig. 11). Over 10% of BAU victims had their trust go down ‘a lot’, double the level of RVR but still not a major change.

### Efficiency

To understand the relative efficiency of RVR, all 517 cases were rigorously monitored and tracked. Digital tracking systems of officers’ cars and radios provided information on response officer movement. CAD records were used to track dispatch decisions and timings of actions taken. All responding officers were asked to complete a follow-up survey in the days after their attendance.

#### Time Taken for the Primary Investigation

Table 2 shows that RVR was 656 times faster than BAU in responding to the abuse victims, and one-third faster than BAU in terms of officer hours consumed in resolving the incident.

**Table 2:** Average overall resolution and response times

	Cases	Average time to resolve	Average time to respond	Average time to resolve (officer minutes – accounts for double crewing)
Business as usual	253	158minutes	1969 minutes	205 minutes
Rapid video response	264	122 minutes	3 minutes	122 minutes

Resolution time is how long the responding officers take dealing with a job. This can be calculated two ways. The first is simply to calculate the time taken to do the job. The second is to work out the time each officer spends at that job, thereby factoring in the number of officers in attendance. If there is only one officer, these two calculations will be the same. But when two officers attend the same job, this doubles the police time it takes to resolve that job. With a combination of double and single crewed responses for business-as-usual cases, this officer time per job averages out to 205 min. As RVR only has a single officer, the resolution time values are identical, at 122 min.

**Fig. 12:**
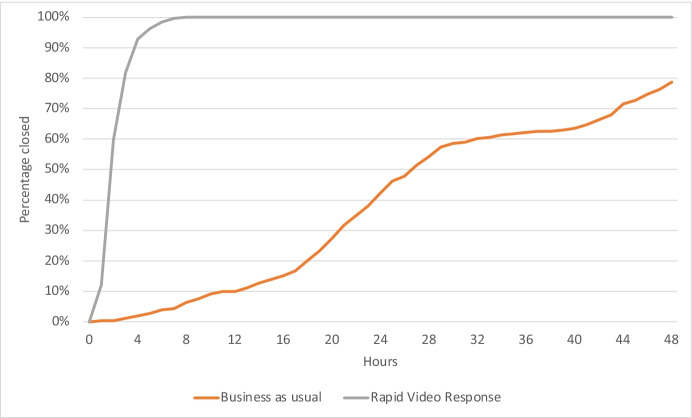
Proportion of RVR and BAU cases having victim contact and resolution over the first 48 h

While the averages are useful to understand a ‘typical’ case, we can also view the percentage of each group that are resolved over time. Figure 12 shows that after 8h, 100% of RVR calls have been completed compared to 7% of business as usual cases. After 48 h, almost 80% of cases in the BAU group have been resolved.

#### Arrests

**Fig. 13:**
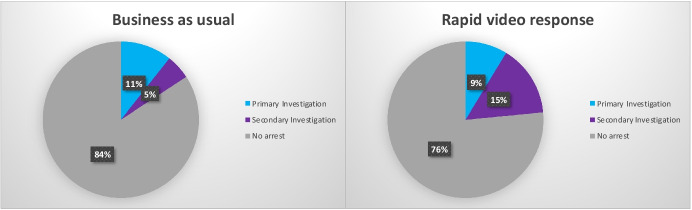
Arrests during stages of RVR and BAU investigations

Figure 13 shows that RVR cases have an arrest rate of 24%, compared to the BAU group at 16%. This difference comes from secondary investigation, where arrests are 3 times more likely to occur on RVR cases than on BAU. There is little difference (11% BAU vs. 9% RVR) during the initial investigation phase.

## Conclusions

The Kent experiment for rapid video response is the first randomised controlled trial of RVR. Given its positive reception by domestic abuse victims, there is ample evidence that it is worth replication in other police agencies. With a primarily white British population, it is possible that these findings depend on having that demographic makeup of the victims requesting police service. Other factors, including travel time, traffic and police force characteristics, could also produce different results. Thus, other forces should probably proceed with caution, but only to the extent of replicating this trial rather than rolling out a new policy without a rigorous impact evaluation.

Even the Kent trial remains uncertain about the most important question of the long-term impact, if any, of RVR on victim harm and future abuse. It would therefore be important for this trial to benefit from at least a 2-year follow-up with all victims and offenders for repeat victimisation, offending and total crime harm index (CHI) (Sherman et al., 2016).

It is interesting that the context of COVID restrictions in which this study was done helped to make it possible, by speeding the development of police video communications capability. At the same time, however, any research on domestic abuse conducted during the 2-year period of intermittent COVID-19 restrictions has a limitation of that context in its generalisability. The restrictions may have increased indoor contact among potential victims and offenders. Conversely, it may have reduced intoxication in public settings that was carried home into private places, thereby reducing various dimensions of domestic abuse. We may never know how or how much the COVID context affected the outcomes, but we can certainly note that further research outside of COVID might reach different conclusions.

One thing that COVID may also have affected is public acceptance of video calls as a means of communicating with police, as well as with everyone else. The rise of work-from-home has normalised video calls in ways that were not seen as likely as late as early 2020. Yet, that normalisation may have enhanced victim preference for an option of calling police to discuss domestic abuse without neighbours seeing a police car parked in front of one’s family home. The current study shows that 75% of eligible victims were willing to accept an offer of a video call, without any preparatory publicity or public education about the option. With the positive findings of this study, that percent might rise even higher in future. It may even encourage more calls to police if victims become aware that they can seek help without risking unwanted local attention.

The evidence in this article clearly provides a strong basis for proceeding with RVR in the force which developed and tested it. It is rare in the history of evidence-based policing that an article reporting the findings of an experiment can also report the impact of the research. This is such a case. On 18th May, 2022, Kent Police launched RVR as a service generally available to appropriate domestic abuse victims who request it anywhere in the County. That appears to be another first as well.

## Figures and Tables

**Fig. 14:**
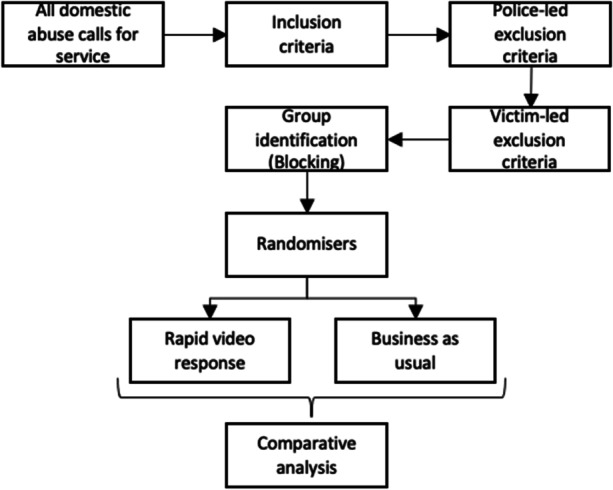
Overview of case-flow and decision pathways

**Fig. 15:**
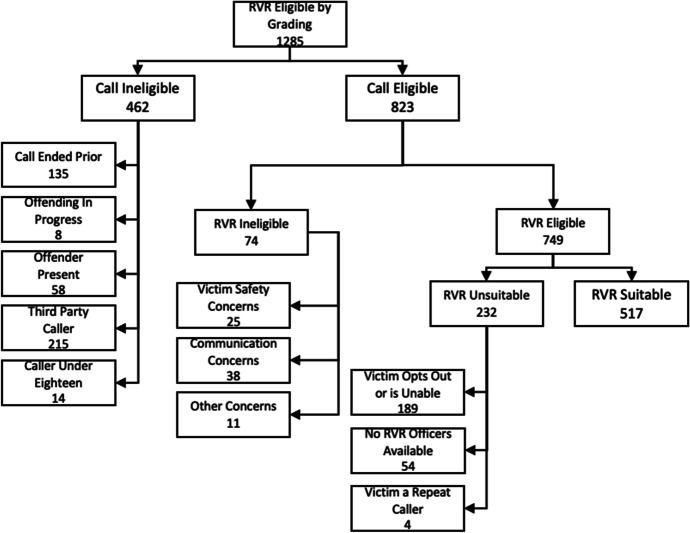
Modified CONSORT diagram of case attrition (See Consolidated Standards of Reporting Trials flow diagram at http://www.consort-statement.org/consort-statement/flow-diagram. Flow process after final sample selection (N = 517) of combined random assignment blocks appears in Fig. 3 above.)

## Appendix 1

### RVR script

The following script was used by police call takers for the RVR trial

‘Due to the high volume of calls we are experiencing; we would like to offer you the opportunity to speak immediately with a police officer, via a recorded video link, to resolve your call. You can use your Android or IOS smartphone, tablet, laptop or computer – there is no need to download an app. This is an optional service that could mean your incident is responded to rapidly. If you are in a suitable and safe location, would you like to speak with an Officer now if one is available?’

If yes:

‘The video relies on the availability of your 3G/4G network to transmit the video stream. If mobile data coverage is not good enough, the stream will not connect or will be of poor quality. You will need sufficient charge on your device or the ability to charge their device during the call. This video call will be recorded, the footage will be owned by the police and the footage can be used as evidence. No footage will be saved to your device. You are free to terminate the call at any point. On termination of the call no further audio or video can obtained from your device. Do you consent to the video call?’

If yes, randomise and either say:

*RVR Treatment* – ‘I will now send a link to your device. You will need to terminate this call with me and agree to the two “GoodSAM” prompts for your consent to the call. If we lose contact - we will call you back after a few minutes on your telephone number. Please take the reference number xxxx.’

*BAU Control* – ‘Unfortunately all of our video officers are currently busy. I will pass your call across to your local area for dispatch.’

## Appendix 2

Figures 14, 15

## Footnotes

1 For RVR, the process of establishing the video link took up to a minute and this time was used to get victim consent and discuss how the victim activates the text message to open the video and speak to the officer. For BAU, this was included prior to the safeguarding advice and advising victims to call police back if circumstances changed prior to police contact.

2 Specifically, it lacks statistical power.
